# L_1_-norm based nonlinear reconstruction improves quantitative accuracy of spectral diffuse optical tomography

**DOI:** 10.1364/BOE.9.001423

**Published:** 2018-03-02

**Authors:** Wenqi Lu, Daniel Lighter, Iain B. Styles

**Affiliations:** 1 School of Computer Science, University of Birmingham, Edgbaston, Birmingham B15 2TT, UK; 2 Physical Sciences for Health Centre for Doctoral Training, University of Birmingham, Edgbaston, Birmingham B15 2TT, UK

**Keywords:** (000.1430) Biology and medicine, (000.4430) Numerical approximation and analysis, (100.3190) Inverse problems, (110.6955) Tomographic imaging

## Abstract

Spectrally constrained diffuse optical tomography (SCDOT) is known to improve reconstruction in diffuse optical imaging; constraining the reconstruction by coupling the optical properties across multiple wavelengths suppresses artefacts in the resulting reconstructed images. In other work, L_1_-norm regularization has been shown to improve certain types of image reconstruction problems as its sparsity-promoting properties render it robust against noise and enable the preservation of edges in images, but because the L_1_-norm is non-differentiable, it is not always simple to implement. In this work, we show how to incorporate L_1_ regularization into SCDOT. Three popular algorithms for L_1_ regularization are assessed for application in SCDOT: iteratively reweighted least square algorithm (IRLS), alternating directional method of multipliers (ADMM), and fast iterative shrinkage-thresholding algorithm (FISTA). We introduce an objective procedure for determining the regularization parameter in these algorithms and compare their performance in simulated experiments, and in real data acquired from a tissue phantom. Our results show that L_1_ regularization consistently outperforms Tikhonov regularization in this application, particularly in the presence of noise.

## 1. Introduction

Diffuse optical tomography (DOT) is a non-invasive, non-ionizing and low-cost imaging technology with applications in diagnosing breast cancer [1–3], analyzing brain function for functional neuroimaging [4–8], and imaging small animals for the study of disease detection, progression/regression and treatment [9,10]. The imaging process typically involves the injection of near-infrared (NIR) light in the spectral range of 650–900nm into the imaging volume of interest (e.g. breast, head, mouse) through sources on the surface of the volume. The transmitted light is then measured at different locations using detectors that are also on the same volume surface. A number of measurements are acquired using different source-detector pairs, and the internal distribution of the tissue’s optical properties is reconstructed using a transport-model-based image reconstruction algorithm [11].

When a single wavelength continuous-wave (CW) light source is used, only the amplitude of the fluence can be measured at the surface boundary. In this case, only tissue absorption at that wavelength can be estimated. However, since the quantities of interest in DOT experiments are typically chromophore concentrations (mainly oxyhemoglobin (HbO_2_) and deoxyhemoglobin (Hb)) rather than the absorption itself, measurements have to be taken at multiple CW wavelengths in order to provide sufficient information to recover the distributions of these chromophores. There are two main approaches for reconstruction of chromophore images using multiple wavelength measurements: non-spectral methods and spectrally constrained methods. Non-spectral methods (traditional DOT) reconstruct the absorption coefficients at each wavelength independently and then calculate the chromophore concentrations using Beer’s law [12]. Spectrally constrained DOT (SCDOT) directly reconstructs the chromophore distributions by using the known absorption spectra of the chromophores to constrain the reconstruction process. Compared with non-spectral methods, spectrally constrained reconstruction has been shown to be better at suppressing artefacts in the resulting reconstructed images and to reduce crosstalk between chromophores and scatter parameters in breast imaging [13–15]. It has also been shown that boundary measurements at two NIR wavelengths are sufficient to recover the concentrations of HbO_2_ and Hb [16].

Reconstruction of images from DOT measurements is a difficult inverse problem. The limited availability of boundary measurements and the diffusive nature of light propagation in tissue [11,17] make the problem non-linear and ill-posed, and iterative solutions with effective methods for regularization are necessary to obtain unique solutions. Many approaches have been used [18–20] and quadratic Tikhonov (L_2_-norm) regularization is the most popular approach as the solutions to each iteration step can be computed analytically, simplifying the reconstruction process. This is known to suppress the high-frequency components of the reconstructed image (normally noise) leading to smooth reconstructions, but this has the drawback of being unable to preserve sharp features in the reconstructed images [19].

L_1_-norm regularization has recently been adopted for single wavelength DOT image reconstruction. Features of interest in DOT, such as tumours in the breast or activations in the brain are typically spatially localized and in this case the vector corresponding to the difference in the optical properties relative to the background is sparse with only a few non-zero elements [21–24]. L_1_-norm regularization is known to induce sparsity in the solution to inverse problems [24–26], and has been shown to give essentially the same sparsity as the true sparsity measure (L_0_-norm) [27]. Compared to L_2_ regularization, L_1_ regularization is less sensitive to outliers, which correspond to sharp edges in image processing applications [28] and is thus able to preserve edge-like features. Both L_2_ and L_1_ regularization methods can be solved by convex optimization schemes where a unique solution can be guaranteed [24, 25]. The more general L*_p_*-norm (0 < *p* < 1) regularization schemes have also been studied for DOT image reconstruction [23,29], and are also known to introduce sparsity to the reconstructed image [30]; however, L*_p_* regularization is known to be nonconvex meaning that local minima exist [31] and unique solutions cannot be guaranteed.

In traditional DOT, L_1_ regularization can indeed be applied to each wavelength independently. However, there are no guarantees that the solutions will be consistent with Beer’s law. It must be assumed that the regularizer will have the same sparsifying effect at all wavelengths. This may not necessarily be true, given that SNR, scattering etc are different. SCDOT can be used to constrain the solution space to those solutions that are physically plausible. Therefore, reconstruction with spatial and spectral regularization simultaneously applied will constrain the solution space much more reliably than their sequential application. To the best of our knowledge, L_1_-norm has not yet been used in SCDOT image reconstruction. We introduce a novel algorithm, spectral-L_1_, which combines the sparsity-preserving advantages of L_1_ regularization with the spectral constraints imposed by coupling optical properties across multiple wavelengths, to solve the inverse problem for image reconstruction in SCDOT. The key advance is to adapt the DOT reconstruction process to incorporate efficient methods for solving each iterative step. These are necessary because the L_1_-norm is non differentiable and the update terms in the reconstruction process cannot be computed analytically. We investigate three algorithms for solving the update term: iteratively reweighted least square algorithm (IRLS) [32], alternating directional method of multipliers (ADMM) [33–36] and fast iterative shrinkage-thresholding algorithm (FISTA) [37,38]. All three methods have been widely used to obtain sparse solutions to linear systems. IRLS and ADMM are second-order algorithms that require explicit inversion of a large matrix; FISTA is a first-order algorithm that does not require explicit matrix inversion, but does require a gradient operator to be constructed.

We adapt the DOT reconstruction process to use these methods for the solution of the update terms. An automated method to automatically select the regularization parameters is developed which is based on the L-curve method but is modified for this use-case. Then we perform a systematic comparison of the different regularization methods (L_1_ and L_2_) and optimization algorithms (IRLS, ADMM and FISTA) on simulated data in two- and three-dimensions. The comparison evaluates the methods on the accuracy of image reconstruction; ability to preserve edges; robustness against noise; and computational efficiency. Comprehensive and robust qualitative and quantitative evaluations are performed to quantitatively compare the reconstruction results using average contrast (AC), Pearson correlation (PC) and peak signal-to-noise ratio (PSNR). To our knowledge, this is the first systematic study in the area of spectral DOT reconstruction to perform such a comprehensive evaluation. We then apply our methods to the reconstruction of functional activations in simulated human brain imaging data using realistic anatomical models and finally evaluate the proposed algorithms using experimentally acquired data, by imaging a tissue-mimicking, plastic phantom of known optical properties using a multispectral DOT system.

The paper is organized as follows: Section 2 introduces the theory for image reconstruction in SCDOT and proposes a new spectral-L_1_ inverse model; Section 3 investigates the performance of the candidate three reconstruction algorithms for our proposed model; In section 4, a principled method for selecting the regularization parameter is described; Section 5 presents extensive comparative experiments in simulated models, and the results of experiments using tissue phantoms. In section 6, the conclusions that can be drawn from our results are discussed.

## 2. Theory

Image reconstruction in SCDOT aims to find the tissue composition that best explains the boundary measurements. It typically requires the repeated evaluation of a forward model of light propagation in biological tissues as part of an inverse model that minimizes the difference between the measurements and the model’s predicted measurements. In this section, the forward model for CW light propagation is introduced, followed by the spectrally constrained inverse model. The L_1_ and L_2_ regularization methods for the inverse problem are described at the end of the section.

### 2.1. The forward model

It is generally accepted that if the scattering coefficients dominate over absorption coefficients in tissues and the region of interest is far from the light sources, light propagation can be modelled by a diffusion equation (DE) [11]. The DE is able to generate isotropic fluence fields given a distribution of source fibres and the tissue optical properties. For a CW system, the DE can be written as

(1) $$-\nabla \cdot \kappa (r,\lambda_{i})\nabla \Phi (r,\lambda_{i})+\mu_{a}(r,\lambda_{i})\Phi (r,\lambda_{i})=q_{0}(r,\lambda_{i}).$$

Here, *μ_a_* (*r*, *λ_i_*) is the absorption coefficient at position *r* for wavelength *λ_i_*, *κ* (*r*, *λ_i_*) = 1/3[*μ_a_*(*r*, *λ_i_*) + $\mu_{s}^{\prime }$(*r*, *λ_i_*)] in which $\mu_{s}^{\prime }$ (*r*, *λ_i_*) is the reduced scattering coefficient. Φ (*r*, *λ_i_*) is the photon density at position *r* and wavelength *λ_i_*, and the isotropic source term at wavelength *λ_i_* is given by *q*_0_ (*r*, *λ_i_*). It should be noted that in CW imaging, the value of $\mu_{s}^{\prime }$ (*r*, *λ_i_*) is not updated by the reconstruction algorithm and is assumed to be a known constant. The air-tissue boundary is represented by an index-mismatched type III condition (Robin or mixed boundary condition) in which the fluence at the edge of the tissue exits and does not return [39, 40]. A finite element method (FEM) is used to numerically solve Eq. (1) on a discretized mesh, which has been implemented in several open-source software packages, notably TOAST++ [41] and NIRFAST [12]. In this work, the NIRFAST package is used for all computations.

In CW systems, the tissue absorption *μ_a_* depends on the concentration of chromophores in the tissue. The relationship between the absorption coefficients at different wavelengths is therefore constrained by the intrinsic absorption properties of the chromophores via Beer’s law. For a dual-wavelength imaging system, and for two chromophores, Beer’s law is written in matrix-vector form as

(2) $$\left(\begin{array}{c} \mu_{a,\lambda_{1}}\\ \mu_{a,\lambda_{2}} \end{array}\right)=\left(\begin{array}{cc} \varepsilon_{c_{1},\lambda_{1}} & \varepsilon_{c_{2},\lambda_{1}}\\ \varepsilon_{c_{1},\lambda_{2}} & \varepsilon_{c_{2},\lambda_{2}} \end{array}\right)\left(\begin{array}{c} c_{1}\\ c_{2} \end{array}\right),$$

where *c*_1_ and *c*_2_ are chromophore concentrations and *λ*_1_ and *λ*_2_ are two measurement wavelengths. In the remainder of the paper *c*_1_ and *c*_2_ correspond to oxyhemoglobin HbO_2_ and deoxyhemoglobin Hb respectively, with *λ*_1_ = 750nm and *λ*_2_ = 850nm. *ε*_*c*_*i*_,*λ*_*i*__ (*i* = 1, 2) are the extinction coefficients of the two chromophores at the corresponding wavelength *λ_i_*. The values of *ε*_*c*_*i*_,*λ*_*i*__ have been documented by Zeff et al (2007) [5].

### 2.2. The inverse model for SCDOT image reconstruction

In SCDOT, chromophore concentrations *c*_1_, and *c*_2_ are directly estimated from the boundary measurements in preference to explicitly reconstructing optical properties at each wavelength. The following SCDOT inverse model allows direct estimation of chromophore parameters from two measurement wavelengths (i.e. 750 and 850nm) using some form of iterative procedure. Using a block notation, in which (∶) represents the concatenation of two column vectors, we have:

(3) $$c_{1},c_{2}=\underset{c_{1},c_{2}}{\text{arg min}}{\left\|\left(\begin{array}{c} \Phi_{\lambda_{1}}^{M}\\ \Phi_{\lambda_{2}}^{M} \end{array}\right)-\left(\begin{array}{c} \Phi_{\lambda_{1}}^{C(k)}\\ \Phi_{\lambda_{2}}^{C(k)} \end{array}\right)\right\|}_{2}^{2},$$

where chromophores *c*_1_ and *c*_2_ are the model parameters to be recovered. $\Phi_{\lambda_{i}}^{M}$ (*i* = 1, 2) is the measured fluence at the tissue surface and $\Phi_{\lambda_{i}}^{C}$ is the calculated data using the forward solver. The superscript *k* denotes the iteration number. Eq. (3) defines a non-linear least square problem which can be solved via the classical Gauss-Newton method in which the first order Taylor series is used to expand the forward solution $\Phi_{\lambda_{i}}^{C}$ as

(4) $$\left(\begin{array}{c} \Phi_{\lambda_{1}}^{C(k)}\\ \Phi_{\lambda_{2}}^{C(k)} \end{array}\right)=\left(\begin{array}{c} \Phi_{\lambda_{1}}^{C(k-1)}\\ \Phi_{\lambda_{2}}^{C(k-1)} \end{array}\right)+J^{k-1}\left(\begin{array}{c} c_{1}^{k}-c_{1}^{k-1}\\ c_{2}^{k}-c_{2}^{k-1} \end{array}\right),$$

in which the spectral Jacobian J (also known as the sensitivity matrix) relates the changes in boundary data to changes in chromophore concentrations and can be constructed directly with the incorporation of spectral prior information using the adjoint method [42]. Note that when *k* = 1, an initial guess of the chromophore concentrations $c_{1}^{0}$ and $c_{2}^{0}$ is required which can be obtained by a data-calibration procedure explained elsewhere [43]. The spectral Jacobian J can be derived as [44]:

(5) $$J=\left(\begin{array}{cc} \frac{\partial \Phi_{\lambda_{1}}^{C}}{\partial c_{1}} & \frac{\partial \Phi_{\lambda_{1}}^{C}}{\partial c_{2}}\\ \frac{\partial \Phi_{\lambda_{2}}^{C}}{\partial c_{1}} & \frac{\partial \Phi_{\lambda_{2}}^{C}}{\partial c_{2}} \end{array}\right)=\left(\begin{array}{cc} \frac{\partial \Phi_{\lambda_{1}}^{C}}{\partial \mu_{a,\lambda_{1}}}\cdot \frac{\partial \mu_{a,\lambda_{1}}}{\partial c_{1}} & \frac{\partial \Phi_{\lambda_{1}}^{C}}{\partial \mu_{a,\lambda_{1}}}\cdot \frac{\partial \mu_{a,\lambda_{1}}}{\partial c_{2}}\\ \frac{\partial \Phi_{\lambda_{2}}^{C}}{\partial \mu_{a,\lambda_{2}}}\cdot \frac{\partial \mu_{a,\lambda_{2}}}{\partial c_{1}} & \frac{\partial \Phi_{\lambda_{2}}^{C}}{\partial \mu_{a,\lambda_{2}}}\cdot \frac{\partial \mu_{a,\lambda_{2}}}{\partial c_{2}} \end{array}\right)=\left(\begin{array}{cc} J_{\lambda_{1}}\cdot \varepsilon_{c_{1},\lambda_{1}} & J_{\lambda_{1}}\cdot \varepsilon_{c_{2},\lambda_{1}}\\ J_{\lambda_{2}}\cdot \varepsilon_{c_{1},\lambda_{2}} & J_{\lambda_{2}}\cdot \varepsilon_{c_{2},\lambda_{2}} \end{array}\right),$$

where J_*λ*_*i*__ relates the changes in boundary data to changes in the absorption coefficient at wavelength *λ_i_*. The size of J in this case is the number of wavelengths times the number of measurements per wavelength, by number of finite element nodes times number of chromophore parameters.

Substituting Eq. (4) into Eq. (3) leads to

(6) $$\Delta c^{k}=\underset{\Delta c}{\text{arg min}}{\left\|\Delta \Phi^{k-1}-J^{k-1}\Delta c\right\|}_{2}^{2},$$

where Δ*c^k^* is the change in the chromophore parameters at the *k*-th iteration and can be written as

(7) $$\Delta c^{k}=\left(\begin{array}{c} \Delta c_{1}^{k}\\ \Delta c_{2}^{k} \end{array}\right)=\left(\begin{array}{c} c_{1}^{k}-c_{1}^{k-1}\\ c_{2}^{k}-c_{2}^{k-1} \end{array}\right).$$

ΔΦ in Eq. (6) is the data-model mismatch which is given by

(8) $$\Delta \Phi^{k-1}=\left(\begin{array}{c} \Phi_{\lambda_{1}}^{M}-\Phi_{\lambda_{1}}^{C(k-1)}\\ \Phi_{\lambda_{2}}^{M}-\Phi_{\lambda_{2}}^{C(k-1)} \end{array}\right).$$

Minimizing Eq. (6) leads to the normal equations

(9) $$\left(J^{(k-1)T}J^{\left(k-1\right)}\right)\Delta c^{k}=J^{(k-1)T}\Delta \Phi^{k-1}.$$

which can be solved to find the update term Δ*c^k^* using the Gauss-Newton algorithm which is summarized in Algorithm 1.

**Algorithm 1:** Gauss-Newton Algorithm for Minimizing Eq. (3).

**INPUT**: $\Phi_{\lambda_{i}}^{M}$ (*i* = 1, 2), *iter*, Tol
**Initialize**: $c_{1}^{0}$, $c_{2}^{0}$
**for** *k* = 1 : *iter*
1: Update *μ*_*a*,*λ*_*i*__ at each wavelength using Beer’s law (Eq. (2))
2: Update $\Phi_{\lambda_{i}}^{C(k-1)}$ at each wavelength using the forward model (Eq. (1))
3: Update ΔΦ^*k*−1^ using Eq. (8)
4: Stop if *k* = *iter* or ‖ΔΦ^*k*−1^ − ΔΦ^*k*−2^‖_1_ ⩽ Tol, otherwise go to step 5
5: Update J^*k*−1^ using Eq. (5)
6: Update Δ*c^k^* = (J^(*k*−1)T^J^(*k*−1)^)^−1^J^(*k*−1)T^ΔΦ^*k*−1^
7: Update $c_{1}^{k}$ and $c_{2}^{k}$ using Eq. (7)
**end for**
**RETURN** $c_{1}^{k}$ and $c_{2}^{k}$

It is however non-trivial to calculate the inverse of J^(*k*−1)T^J^(*k*−1)^ in Eq. (9) (i.e. step 6 in Algorithm 1) because it is normally singular or close to singular. Furthermore, experimental noise in the measurements $\Phi_{\lambda_{i}}^{M}$ tends to lead to reconstruction artefacts if this inversion is computed directly. Strategies that can be employed to invert such ill-posed matrices includes algebraic reconstruction technique (ART), truncated singular value decomposition (TSVD) or the simultaneous iterative reconstruction technique (SIRT) [1,11,45–47]. Regularization can also be employed to reduce model errors and artefacts caused by measurement noise. In the following section, we introduce an L_1_-based regularization technique to solve this ill-posed inverse problem.

### 2.3. The proposed spectral-L_1_ inverse model

To convert Eq. (6) into a more readily solvable problem, a Tikhonov (L_2_) regularization term is usually introduced into the inverse problem:

(10) $$\Delta c^{k}=\underset{\Delta c}{\text{arg min}}\left\{{\left\|\Delta \Phi^{k-1}-J^{k-1}\Delta c\right\|}_{2}^{2}+\lambda {\left\|\Delta c\right\|}_{2}^{2}\right\}.$$

The regularization parameter *λ* determines the degree of regularization that will be imposed on the model. This can be solved analytically to give

(11) $$\Delta c^{k}={\left(J^{(k-1)T}J^{\left(k-1\right)}+\lambda I\right)}^{-1}J^{(k-1)T}\Delta \Phi^{k-1}.$$

I is the identity matrix and its size is the same as that of J^(*k*−1)T^J^(*k*−1)^. The introduction of *λ*I effectively reduces the condition number of the matrix, thus stabilizing the matrix inversion. This is widely known as the Levenberg-Marquardt algorithm and is in general more robust than the Gauss-Newton method. An analytical solution to this problem is possible because Eq. (10) is convex and quadratic which makes L_2_-norm regularization an attractive choice for many inverse problems. However, in image reconstruction problems, it tends to over-smooth the result and sharp features such as object boundaries in the reconstructed images are often smeared. Moreover, L_2_-norm discourages sparsity, and is not suitable for sparse image reconstruction. In SCDOT image recovery, the perturbation/change Δ*c^k^* is usually zero or close to zero when the region to be recovered is not in the vicinity of the region of interest. In this case, Δ*c^k^* is spatially sparse. Recently, L_1_ regularization has been widely studied because of several useful properties: it is sparsity-promoting, convex, edge-preserving, and is more robust against noise. Therefore, we propose a new inverse model for SCDOT image recovery based on L_1_ regularization that we refer to as spectral-L_1_. This is formulated as

(12) $$\Delta c^{k}=\underset{\Delta c}{\text{arg min}}\left\{{\left\|\Delta \Phi^{k-1}-J^{k-1}\Delta c\right\|}_{2}^{2}+\lambda {\left\|\Delta c\right\|}_{1}\right\}.$$

Although L_1_ regularization has many advantages over L_2_ regularization, the L_1_-norm is non-differentiable, which makes it difficult to solve Eq. (12). Three candidate algorithms for this task will be investigated in the next section.

## 3. Candidate algorithms for solving the proposed spectral-L_1_ method

We now consider three algorithms for the solution of Eq. (12): iteratively reweighted least squares (IRLS) [32]; alternating directional method of multipliers (ADMM) [33]; and fast iterative shrinkage-thresholding algorithm (FISTA) [37]. These algorithms will be incorporated into the image reconstruction process by substituting them into step 6 of Algorithm 1, which solves for the update term.

### 3.1. IRLS

Instead of solving the L_1_-minimization problem directly, IRLS reformulates the problem as a sequence of weighted L_2_ minimization problems. Specifically, by introducing a weight matrix W, the L_1_ minimization can be converted into finding the optimal solution of the quadratic problem

(13) $$\Delta c^{k}=\underset{\Delta c}{\text{arg min}}\left\{{\left\|\Delta \Phi^{k-1}-J^{k-1}\Delta c\right\|}_{2}^{2}+\lambda {\left\|W\Delta c\right\|}_{2}^{2}\right\}.$$

W is a diagonal matrix with weights, *w_s_*, along its diagonal that are given by

(14) $$w_{s}=\{\begin{array}{ll} {\left|\Delta c_{s}^{i-1}\right|}^{-0.5} & \text{if}\;\left|\Delta c_{s}^{i-1}\right|⩾\varepsilon \\ \varepsilon^{-1} & \text{if}\;\left|\Delta c_{s}^{i-1}\right|<\varepsilon \end{array}.$$

The superscript *i* above denotes the *i*’th IRLS iteration (Algorithm 2), and it should be distinguished from the superscript *k* denoting the iterations of Algorithm 1. A small positive number 0 < *ε* ≪ 1 is used to avoid the possibility of division by zero. It has been suggested that *ε* should be a series of positive real numbers that decay to zero over iterations [48]. In practice, we have found that using a fixed value in the range 0.001 ≤ *ε* ≤ 0.01 does not give significantly different results. Eq. (13) results in the normal equation

(15) $$\left(J^{(k-1)T}J^{\left(k-1\right)}+\lambda W^{T}W\right)\Delta c=J^{(k-1)T}\Delta \Phi^{k-1}.$$

This is known as the weighted L_2_-minimization scheme. We note that if the diagonal weights *w_s_* are set to 1, the normal equation reduces to the conventional L_2_-minimization scheme (Eq. (11)). The IRLS algorithm employing this method is summarized in Algorithm 2.

**Algorithm 2:** Iteratively Reweighted Least Square Algorithm (IRLS)

**INPUT**: ΔΦ^*k*−1^, J^*k*−1^, *λ*, *iter*, Tol
**Initialize**: Set Δ*c*^0^ using Eq. (11)
**for** *i* = 1 : *iter*
1: Update W using Eq. (14)
2: Update Δ*c^i^* = (J^(*k*−1)T^J^(*k*−1)^ + *λ*W^T^W)^−1^J^(*k*−1)T^ΔΦ^*k*−1^
3: Stop if *i* = *iter* or ‖Δ*c^i^* − Δ*c*^*i*−1^‖_1_ ⩽ Tol, otherwise go to step 1
**end for**
**RETURN** Δ*c^k^* = Δ*c^i^*

The calculation of the elements of W requires an initial guess for Δ*c* for which we use the solution to the L_2_-regularized problem, Eq. (11). One of the biggest advantages of IRLS is that Eq. (15) has an analytical solution which allows Eq. (13) to be solved exactly, making IRLS almost as easy to implement as the Levenberg-Marquadt scheme. In common with many sparsity-promoting optimization methods, the sparsity level in IRLS is controlled by the regularization parameter *λ* which must be chosen carefully for each specific problem.

### 3.2. ADMM

ADMM has been widely used to solve optimization problems in machine learning, signal processing, and standard image restoration and reconstruction. This method has become particularly important in the field of variational image processing, which frequently requires the minimization of non-differentiable objectives [33,34,49,50]. It has been shown to be able to solve constrained optimization problems effectively and efficiently. The basic idea is to decompose a complex optimization problem into several simpler subproblems, which usually have closed-form solutions [35]. Its simplicity, flexibility, and broad applicability have made it an important part of the modern optimization toolset. To apply ADMM to our spectral-L_1_ problem, we first introduce an auxiliary splitting vector variable *v*, an augmented Lagrangian multiplier *b*, and a positive penalty parameter *θ*, reformulating Eq. (12) as the following unconstrained optimization problem

(16) $$\Delta c,v,b=\underset{\Delta c,v,b}{\text{arg min}}\left\{{\left\|\Delta \Phi^{k-1}-J^{k-1}\Delta c\right\|}_{2}^{2}+\lambda {\left\|v\right\|}_{1}+\frac{\theta }{2}{\left\|v-\Delta c-b\right\|}_{2}^{2}\right\}.$$

This multivariate optimization problem corresponds to a sub-minimization problem with respect to Δ*c*, *v* and *b*, separately. When all the subproblems converge, the solution for Δ*c* approximately represents that of Eq. (12). In order to find the minimizers for all of the subproblems, ADMM searches all the saddle points of Eq. (16) by first fixing the variables (*v*, *b*) and minimizing the subproblem with respect to Δ*c* using the following normal equation

(17) $$\left(J^{(k-1)T}J^{\left(k-1\right)}+\theta I\right)\Delta c=J^{(k-1)T}\Delta \Phi^{k-1}+\theta (v^{i-1}-b^{i-1}).$$

By inverting the matrix on the left-hand side of Eq. (17), a unique solution for Δ*c* is found. We then fix variables Δ*c* and *b* and set the first order derivative with respect to *v* to zero. This leads to

(18) $$\lambda \frac{v}{\left|v\right|}+\theta \left(v-\Delta c^{i}-b^{i-1}\right)=0,$$

which can be solved component-wise using an analytical shrinkage-thresholding method to give

(19) $$v^{i}=\text{max}\left(\left|\Delta c^{i}+b^{i-1}\right|-\frac{\lambda }{\theta },0\right)○\mathrm{sign}\left(\Delta c^{i}+b^{i-1}\right),$$

where ○ and *sign* symbols denote component-wise multiplication and the signum function, respectively. The last step of ADMM is to update the augmented Lagrangian multiplier *b*, as *b^i^* = *b*^*i*−1^ + Δ*c^i^* − *v^i^*. The complete method is presented in Algorithm 3. The key advantage of ADMM is that Eqs. (17) and (18) have closed-form solutions. We note that the augmented Lagrangian multiplier means that different choices of the penalty parameter *θ* will provide similar results but with different rates of convergence. In all the experiments we have conducted, we used *θ* = 0.01 to achieve fast convergence.

**Algorithm 3:** Alternating Directional Method of Multipliers (ADMM)

**INPUT**: ΔΦ^*k*−1^, J^*k*−1^, *λ*, *θ*, *iter*, Tol
**Initialize**: *v*^0^ = 0, *b*^0^ = 0
**for** *i* = 1 : *iter*
1: Update Δ*c^i^* = (J^(*k*−1)T^J^(*k*−1)^ + *θ*I)^−1^(J^(*k*−1)T^ΔΦ^*k*−1^ + *θ*(*v*^*i*−1^ − *b*^*i*−1^))
2: Update *v^i^* using Eq. (19)
3: Update *b^i^* = *b*^*i*−1^ + Δ*c^i^* − *v^i^*
4: Stop if *i* = *iter* or ‖Δ*c^i^* − Δ*c*^*i*−1^‖_1_ ⩽ Tol, otherwise go to step 1
**end for**
**RETURN** Δ*c^k^* = Δ*c^i^*

### 3.3. FISTA

FISTA is a very efficient optimization approach that uses the forward-backward splitting technique (FBS) [37,38]. It is an extension of the classical gradient descent method and therefore belongs to the class of first order methods that are a better choice for large-scale problems than second-order methods such as IRLS and ADMM because they do not require the explicit construction of very large matrices. Let us consider minimizing the L_1_-regularized data fitting energy given by Eq. (12). We begin by analyzing the standard unregularized problem with *λ* = 0. Let $F(\Delta c)={\left\|\Delta \Phi^{k-1}-J^{k-1}\Delta c\right\|}_{2}^{2}$ and ∇F(Δ*c*) = J^(*k*−1)T^(J^(*k*−1)^Δ*c* − ΔΦ^*k*−1^) denote its gradient. We apply the gradient descent algorithm

(20) $$\Delta c^{i}=\Delta c^{i-1}-t\nabla F\left(\Delta c^{i-1}\right),$$

where *t* > 0 is a suitable stepsize which controls how far the iteration moves along the gradient direction in the *i*’th iteration. The value of *t* is initialized by estimating the Lipschitz constant *L̃* of ∇F as *L̃* = *L* (∇F) and then backtracking rules are adopted to guarantee that the objective has decreased sufficiently [38]. The gradient iteration given by Eq. (20) can be understood as a proximal regularization [51] of the linearized function F(Δ*c*) at Δ*c*^*i*−1^, which corresponds to the following optimization problem:

(21) $$\Delta c=\underset{\Delta c}{\text{arg min}}\left\{F\left(\Delta c^{i-1}\right)+\nabla F\left(\Delta c^{i-1}\right)\left(\Delta c-\Delta c^{i-1}\right)+\frac{1}{2t}{\left\|\Delta c-\Delta c^{i-1}\right\|}_{2}^{2}\right\}.$$

Analogously, adopting the same gradient descent idea to solve Eq. (12) with *λ* ≠ 0 leads to the following minimization problem

(22) $$\Delta c=\underset{\Delta c}{\text{arg min}}\left\{\frac{1}{2t}{\left\|\Delta c^{i-1}-t\nabla F\left(\Delta c^{i-1}\right)-\Delta c\right\|}_{2}^{2}+\lambda {\left\|\Delta c\right\|}_{1}\right\}.$$

Minimizing this results in a formulation similar to Eq. (18) and can be solved in the same way to give

(23) $$\Delta c^{i}=\text{max}\left(\left|\Delta c^{i-1}-t\nabla F\left(\Delta c^{i-1}\right)\right|-t\lambda ,0\right)○\mathrm{sign}\left(\Delta c^{i-1}-t\nabla F\left(\Delta c^{i-1}\right)\right).$$

The minimizer of the Eq. (12) can then be found by iterating Δ*c^i^* in Eq. (23) to convergence. In isolation, Eq. (23) is known as the iterative shrinkage-thresholding algorithm (ISTA) [52–57], whose global convergence rate is *O*(1/*N*), where *N* is the iteration counter. This is improved upon by using a Nesterov-type acceleration technique to obtain faster convergence. In the FISTA algorithm, the iterative shrinkage operator is not used on the value obtained from the previous iteration Δ*c*^*i*−1^, but rather on a combination of the values from the previous two iterations. Thus, in FISTA, Eq. (23) is replaced with

(24) $$\Delta c^{i}=\text{max}\left(\left|\Delta y^{i}-t\nabla F\left(\Delta y^{i}\right)\right|-t\lambda ,0\right)○\mathrm{sign}\left(\Delta y^{i}-t\nabla F\left(\Delta y^{i}\right)\right),$$

where Δ*y^i^* comes from the prediction procedure given in step 4 of Algorithm 4. This step can help to push the solution to the current iteration further in the direction it moved during the previous iteration, which can significantly improve the computational efficiency. The complete FISTA method is presented in Algorithm 4, where steps 2 and 3 implement the acceleration strategy and can be viewed as an over-relaxation step that improves the global convergence rate from *O*(1/*N*) to *O*(1/*N*^2^).

**Algorithm 4:** Fast Iterative Shrinkage-Thresholding Algorithm (FISTA)

**INPUT**: ΔΦ^*k*−1^, J^*k*−1^, *λ*, *iter*, Tol
**Initialize**: Set Δ*c*^0^ using Eq. (11), *α*^0^ = 1, *t* < 1/*L̃*
**for** *i* = 1 : *iter*
1: Update Δ*c^i^* using Eq. (24)
2: Stop if *i* = *iter* or the relative residual ⩽ Tol, otherwise go on to step 3
3: Update $\alpha^{i}=\left(1+\sqrt{1+4{\left(\alpha^{i-1}\right)}^{2}}\right)/2$
4: Update Δ*y*^*i*+1^ = Δ*c^i^* + (*α*^*i*−1^ − 1)/(*α^i^*)(Δ*c^i^* − Δ*c*^*i*−1^)
**end for**
**RETURN** Δ*c^k^* = Δ*c^i^*

### 3.4. The spectral-L_1_ algorithm

**Fig. 1:**
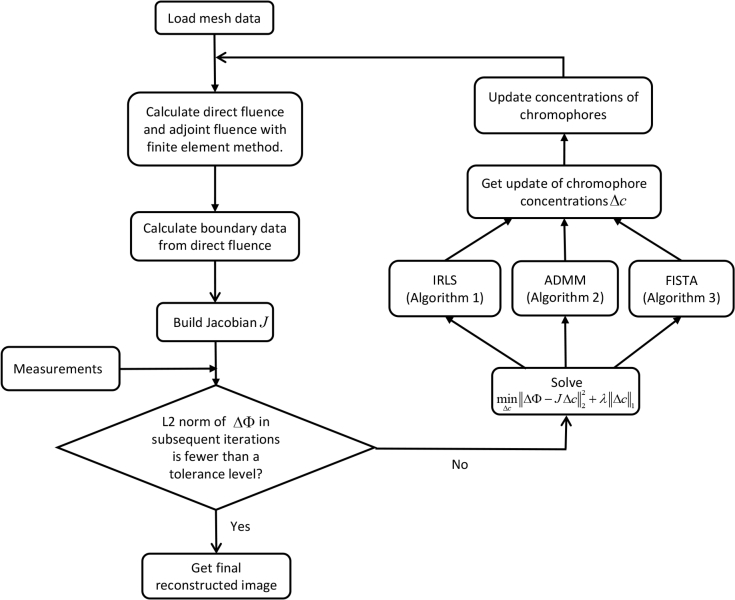
Flow chart for SCDOT image reconstruction using the proposed spectral-L_1_ model.

We have introduced three methods for solving the chromophore update terms in SCDOT with a sparsity enforcing constraint: IRLS, ADMM, and FISTA. These algorithms replace the single update term of the conventional reconstruction algorithm (step 6 of Algorithm 1). The flow-chart presented in Fig. 1 shows how these proposed methods are integrated into SCDOT reconstruction for CW imaging. Since the three proposed optimization schemes are themselves iterative, our method contains nested iterations. In IRLS and FISTA, an initial guess for Δ*c* is required. We use the standard L_2_-regularized solution (Eq. (11)). This is only required on the first iteration of the outer loop.

## 4. Parameter selection

**Fig. 2:**
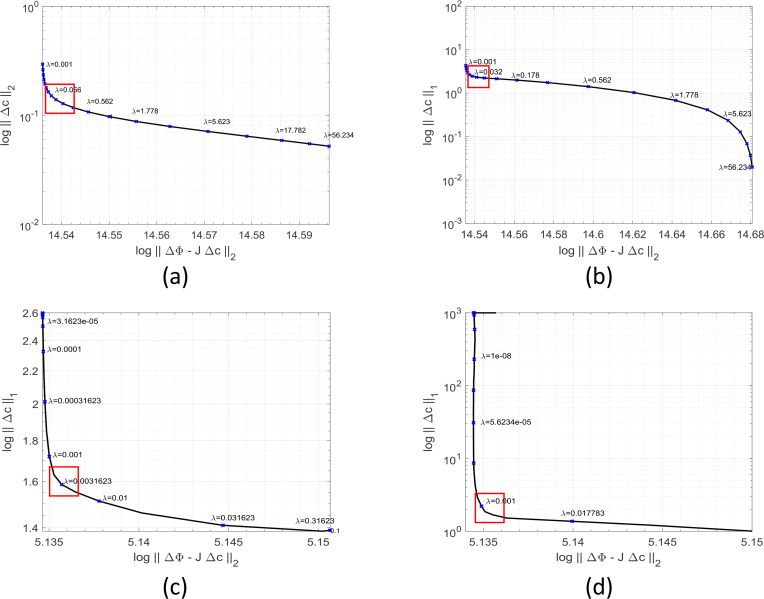
L-curves (data fit against model regularization) derived from a synthetic example: a) Tikhonov regularization; b) L_1_ regularization using the IRLS algorithm; c) L_1_ regularization using the ADMM algorithm; d) L_1_ regularization using the FISTA algorithm. The optimal regularization parameter is around the point of maximum curvature (within the red boxes).

The regularization parameter *λ* determines the trade-off between the goodness-of-fit of the model to the data, and the strict enforcement of the regularization criteria. An optimal value between the two quantities must therefore be found: if too much regularization is imposed on the model, then it will not fit the data properly; if the regularization parameter is too small, the fit will be good but the solution will be dominated by data errors and measurement noise (the overfitting regime). There are several methods to find an optimal compromise between these two considerations and the L-curve method is both simple and effective. By plotting the model-data mismatch ${\left\|\Delta \Phi -J\Delta c\right\|}_{2}^{2}$ against the model regularization ${\left\|\Delta c\right\|}_{2}^{2}$ or ‖Δ*c*‖_1_ for a sequence of different *λ*, a curve which is typically L-shaped can be constructed. Figure 2 shows the L-curves obtained from each of the four candidate optimization schemes using the numerical experiments described by Zhan et al [44]. The optimal trade-off occurs at the “elbow” of the L-shaped curve and this can be located by determining the point of maximum curvature of the curve.

Since strong regularization can improve the conditioning of the linear system, we solve the formulations given by Eqs. (10) and (12) with a relatively large regularization parameter *λ* and then decrease it gradually by a fixed factor until the curvature of the L-curve starts to decrease. This corner point is considered to be at the optimum value of *λ* where both the model fit and the regularization function are simultaneously near to their minimum values. In principle, computing the L-curve requires the full image reconstruction process to be run multiple times which is computationally very expensive. We have found that it is sufficient to compute the L-curve for one iteration of the outer loop of Fig. 1, and then to use the resulting optimal value of *λ* for the remaining iterations. In addition, in order to avoid the special case where the L-curve does not allows an optimal value of *λ* to be found by purely numerical means [58, 59], we select a range around the parameter with the highest curvature value. We then adjust the values manually to get the final optimal parameter by visually inspecting the solutions and choosing the one that generates the sparsest solution with a well-defined compact localization. This approximate optimum is then used for subsequent iterations. We note that the choice of algorithm for L_1_ regularized reconstruction significantly affects the shape of the L-curve and the optimal value of *λ*.

In addition to the regularization parameter *λ* that is common to all three L_1_ algorithms, we have considered how to select the other parameters of each method to ensure that our comparison is fair and unbiased. IRLS has one parameter *ε* and we set this to 0.001 ≤ *ε* ≤ 0.01 following the recommendations set out by Shaw and Yalavarthy [48]. ADMM has one parameter *θ* and the use of the augmented Lagrangian multiplier means that different choices of *θ* provide similar results but lead to different rates of convergence. In all the experiments, *θ* was set to 0.01 to achieve fast convergence. FISTA has two parameters *t* and *α*. *t* is initialized by estimating the Lipschitz constant and then backtracking rules are adopted to guarantee that the objective has decreased sufficiently [38]. *t* is therefore updated automatically. *α* is involved in an over-relaxation step (i.e. step 4 in Algorithm 3) and its update is also automatic (i.e. step 3 in Algorithm 3). The regularization parameter *λ* is therefore the only parameter that must be optimized for a specific problem.

## 5. Experiment setup

We have performed extensive experiments to evaluate the performance of different models and algorithms qualitatively and quantitatively. We first define three evaluation metrics to quantify the quality of the reconstructed images. We then describe simulated numerical experiments, and then real experiments performed on phantom samples. For experiments in which measurement noise was added, ten repeats were performed. In all cases, the forward model was implemented using the NIRFAST package [12] in Matlab R2013a (Mathworks, Natick, USA).

### 5.1. Quantitative evaluation metrics

Three quantitative evaluation metrics are considered: the average contrast (AC), Pearson correlation (PC) and peak signal-to-noise ratio (PSNR). Ideally, if the reconstructed image is exactly same as the ground truth image, AC is equal to 1. For PC and PSNR, the recovered image has higher quality if higher PC or PSNR values are obtained.

Average constrast (AC) is based on the mean value of the region of interest and is defined as:

(25) $$\text{AC}=\frac{\sum_{j=1}^{N}c_{i}^{j}/N}{{\tilde{c}}_{i}}\;i=1,2$$

where $c_{i}^{j}$ denotes the recovered values of chromophore *i* on the finite element node *j*. *N* is the number of nodes in the activation region which is selected by thresholding the recovered changes based on 50% of the maximum recovered changes. *c̃_i_* are the ground truth values of the chromophores in the activation region.

The second evaluation metric PC is given by

(26) $$\text{PC}=\frac{\text{COV}(c_{i},{\tilde{c}}_{i})}{\sigma (c_{i})\sigma ({\tilde{c}}_{i})}\;i=1,2.$$

The numerator is the covariance (COV) of the recovered images with the ground truth and *σ* indicates standard deviation. The PC is thus a measure of the joint variability of the ground truth image with the reconstructed image.

Finally, PSNR evaluates the difference between the ground truth image and the recovered image. Larger PSNR values means less difference between the target and the recovered image. This measure is defined as follows

(27) $$\text{PSNR}=10\cdot {\text{log}}_{10}\left(\frac{{\text{MAX}}_{c_{i}}^{2}}{\text{MSE}}\right)\;i=1,2.$$

Here, MAX_*c*_*i*__ is the maximum pixel value of *c_i_* and MSE is the mean squared error between the reconstructed and ground truth values.

(28) $$\text{MSE}=\frac{1}{N}\sum_{j=1}^{N}{\left(c_{i}^{j}-{\tilde{c}}_{i}^{j}\right)}^{2}$$

For AC, values closer to 1 indicate better performance. For PC and PSNR, higher values are better.

### 5.2. Three-dimensional head numerical experiments

We first evaluate our proposed methods using a physically realistic three dimensional head model derived from T1-weighted MPRAGE scans originally acquired by Eggebrecht *et al* [8] that were kindly provided to us by the authors of that work. Following the process described by Wu et al [60], Statistical Parametric Mapping (SPM) software [61] was used to perform a parametric segmentation of the 5 tissue types (scalp, skull, cerebrospinal fluid (CSF), gray matter, white matter) based on the pixel intensity probability function distribution. These five different layers were then further processed in NIRFAST to create masks and layered volumetric FEM meshes.

**Fig. 3:**
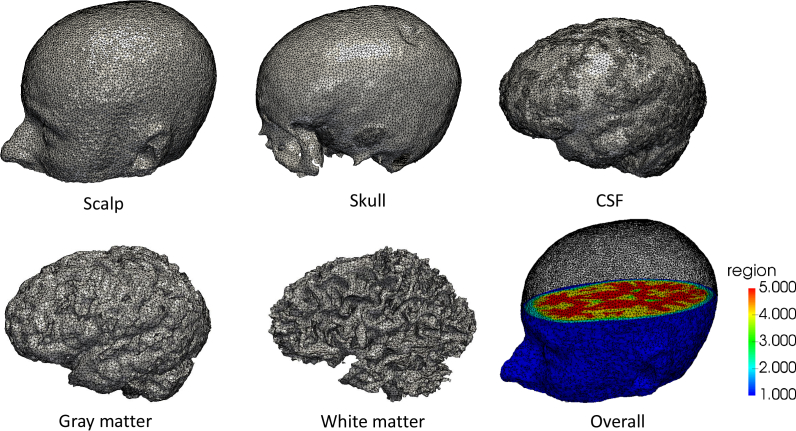
Three-dimensional surface mesh for each of the five head layers.

**Fig. 4:**
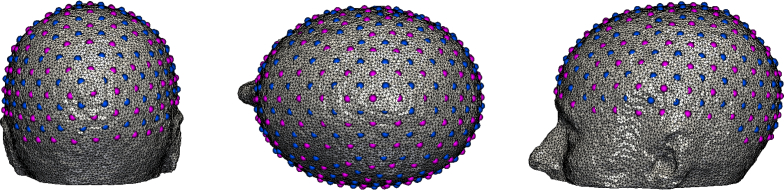
Schematic view from three directions showing the distribution of the imaging array with 158 sources (blue circles) and 166 detectors (red circles).

**Fig. 5:**
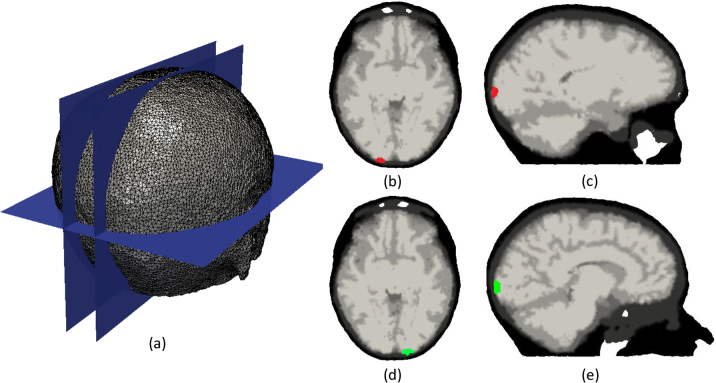
Ground-truth image with the activation only exists in the gray matter and white matter. (a): Illustration of the overall distribution of slices. (b)–(c): Individual activation is color-coded in red and represents the individual simulation of HbO_2_. (d)–(e): Individual activation is color-coded in green and represents the individual simulation of Hb.

The mesh is composed of 101046 nodes corresponding to 589658 tetrahedral elements. Each node is labelled by one of the five segmented head tissue types, as shown in Fig. 3. Chromophore concentrations assigned to each layer are computed from the tissue optical properties at 750nm and 850nm in a previous in vivo study [62] (Table 1) using Beer’s law and Mie scattering formulae. A high-density (HD) imaging array containing 158 sources and 166 detectors (Fig. 4) [8] was placed over the whole head, with source-detector (SD) separation distances ranging from 1.3 to 4.8cm. In this study, 3478 differential measurements per wavelength were used to image the hemodynamic changes in the brain. Two individual activations were simulated in the visual cortex with chromophore concentrations of HbO_2_ and Hb respectively increasing to double the background level in the gray matter (Fig. 5). Each simulated activation has a radius of 5mm. 0% to 2% distributed Gaussian noise at 0.5% intervals was added to the measurement vector.

**Table 1:** Head tissue optical property for each of five layers.

	Scalp	Skull	Cerebrospinal Fluid(CSF)	Gray Matter	White Matter
*c*_1_ (mM)	0.0575	0.0438	0.011	0.0548	0.0683
*c*_2_ (mM)	0.0313	0.0209	0.0083	0.0354	0.0273
Scattering amplitude	0.53	0.7258	0.3	0.5040	0.8176
Scattering power	1.1599	0.8987	0.9*e*^−6^	1.7757	1.3048
*μ_a_* (mm^−1^) at 750nm	0.017	0.012	0.004	0.018	0.017
$\mu_{s}^{\prime }$ (mm^−1^) at 750nm	0.74	0.94	0.3	0.84	1.19
*μ_a_* (mm^−1^) at 850nm	0.019	0.014	0.004	0.019	0.021
$\mu_{s}^{\prime }$ (mm^−1^) at 850nm	0.64	0.84	0.3	0.6726	1.0107

**Fig. 6:**
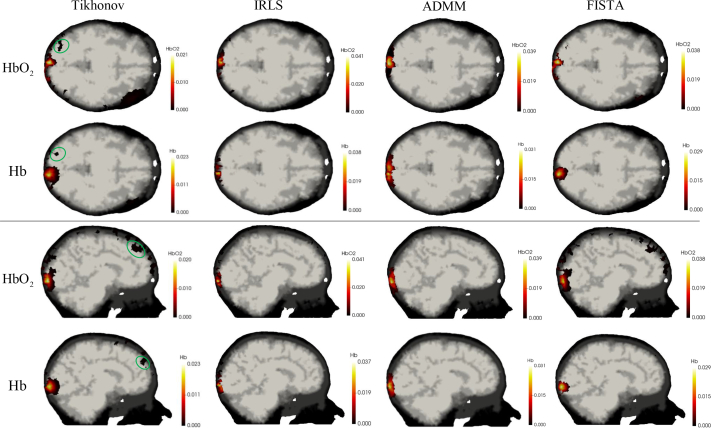
The reconstructed image of the change of HbO_2_ and Hb in mM with noise-free data. Some examples of reconstruction artefacts are highlighted in green ellipses.

**Fig. 7:**
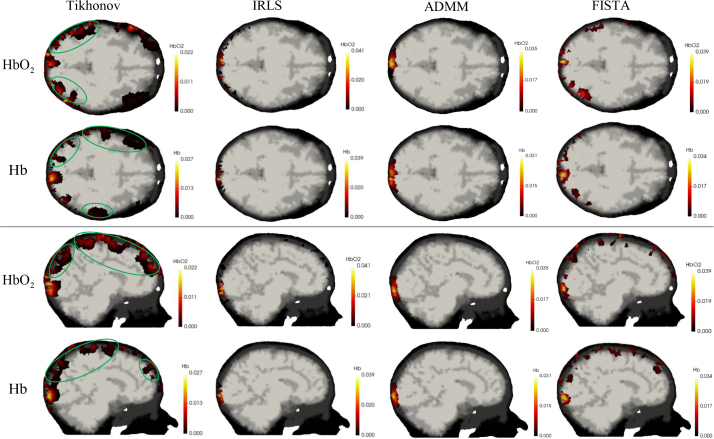
The reconstructed image of the change of HbO_2_ and Hb in mM with data contaminated by 1% Gaussian noise. Some examples of reconstruction artefacts are highlighted in green ellipses.

Reconstructed chromophore concentrations of the simulated activation using the Tikhonov model (Eq. (10)) and the spectral-L_1_ model (Eq. (12)) on noise-free data are displayed in Fig. 6, while those on data with 1% Gaussian noise are displayed in Fig. 7. We only show the area with changes in chromophore concentration greater than 0.0001mM. Compared to the results from the spectral-L_1_ model, Tikhonov reconstructions have lower image contrast, which can be clearly seen from the first column of Fig. 6 and Fig. 7. Some artifacts (areas contained within green ellipses) can be easily observed around the source and detector areas. With increased levels of noise, larger artefacts are seen with Tikhonov regularization and the results are spatially smeared. In contrast, results from the spectral-L_1_ model show fewer artifacts in the non-activation area. Higher noise does not noticeably affect the L_1_-regularized reconstructions. IRLS produces visually more compact localizations than ADMM and FISTA, whilst ADMM appears to have better sparsity-inducing properties compared with IRLS and FISTA.

**Fig. 8:**
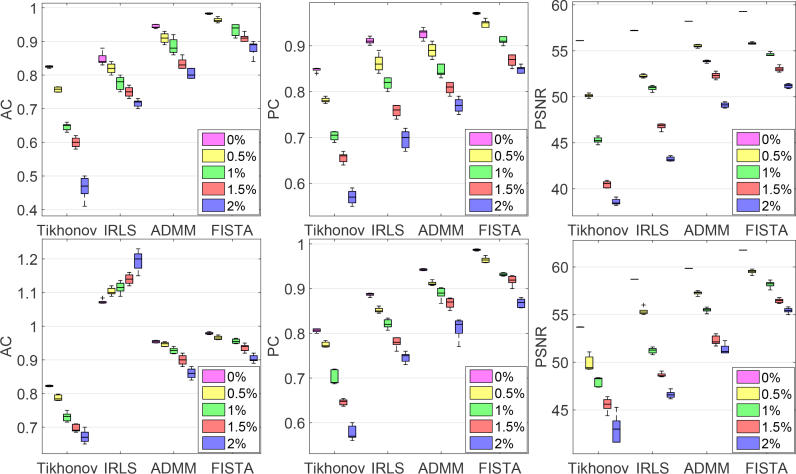
Evaluation metrics comparing the performance of different methods on a simulated 3D head model at five different noise levels. Left to right column: AC index; PC index and PSNR index. The first row gives the results from HbO_2_; the second row from Hb.

Evaluation metrics from this experiments are shown in Fig. 8. It is clear that the spectral-L_1_ model can achieve higher AC, PC and PSNR values than the Tikhonov model which means higher image contrast and accuracy can be achieved with L_1_ regularization. Although the results of FISTA show more visual artifacts than other L_1_-norm methods, it is still able to produce better performance based on the metrics. This is because (i) AC is defined on the activation region which is selected by thresholding the recovered changes based on 50% of the maximum recovered changes, artefacts away from this region do not influence this metric; (ii) By the other metrics (PC and PSNR), the improved ability of FISTA to localize the activation is sufficient to counteract the effect of the artefacts.

### 5.3. More realistic three dimensional head numerical experiments

**Fig. 9:**
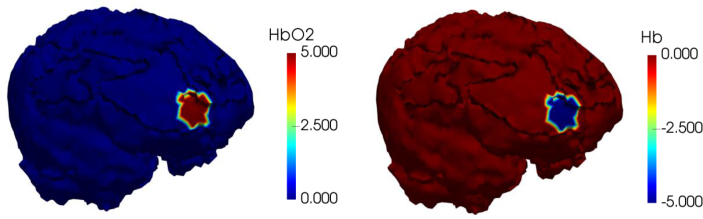
Ground-truth image showing the change in chromophore concentration confined to the gray matter.

Following the proof-of-concept experiments described in the previous section, we extended our analysis to a more realistic case with much smaller changes in chromophore concentrations. In the activation area we model a small region with changes in HbO_2_ (*c*_1_) of 5*μ*M and Hb (*c*_2_) of −5*μ*M, relative to the background concentrations given in Table 1 (Fig. 9). The mesh is the same as that used in the previous section. In line with the expected in vivo performance of imaging systems, 0.12%, 0.15%, 0.41% and 1.42% Gaussian random noise was added to first (13mm), second (30mm), third (40mm) and fourth (48mm) nearest neighbour measurements to provide realistic data [63].

**Fig. 10:**
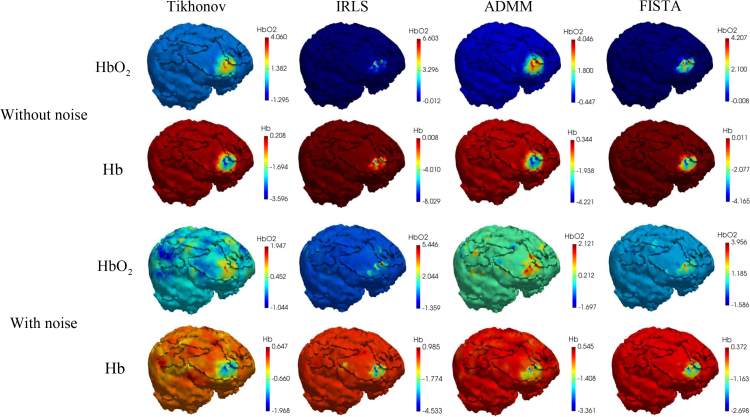
Reconstruction of HbO_2_ and Hb using (L–R): Tikhonov for L_2_-norm regularization; IRLS, ADMM, FISTA algorithms for L_1_-norm regularization with different noise levels. First two rows: results with clean simulated data; Last two rows: those with noisy data.

Reconstruction using the four methods considered here are shown in Fig. 10 with noise-free and noisy simulated data. With reference to results shown earlier in this paper, we make a similar observation that in comparison to the ground truth values, results using Tikhonov regularization are visually inferior to those from L_1_ regularization. With increased noise, Tikhonov regularization performs progressively worse with more artefacts visible in the source-detector areas. L_1_ regularization induces sparse results with fewer artefacts in non-activated areas. Visual inspection of the results from the three L_1_ algorithms suggests that IRLS produces over-sparse reconstructions with strong activations confined to a small area. ADMM and FISTA results are much more visually realistic and they are seen to give higher quantitative accuracy. A quantitative evaluation using AC, PC and PSNR is given in Table 2 and Table 3 and these support the conclusion that even at small changes in chromophore concentration, the spectral-L_1_ model can still guarantee higher image contrast and accuracy, with FISTA performing consistently better by all measures (AC closer to 1, higher PC, higher PSNR).

**Table 2:** Three evaluation metrics for HbO_2_ on results by different methods

	Without noise				With noise			
	Tikhonov	IRLS	ADMM	FISTA	Tikhonov	IRLS	ADMM	FISTA
AC	0.7180	0.8132	0.8367	**0.9069**	0.6674	0.7188	0.7294	**0.7556**
PC	0.9997	0.9998	0.9998	**0.9999**	0.9973	0.9996	0.9996	**0.9998**
PSNR	73.8062	76.4843	77.3333	**78.5520**	38.082	63.7853	67.9154	**73.4270**

**Table 3:** Three evaluation metrics for Hb on results by different methods

	Without noise				With noise			
	Tikhonov	IRLS	ADMM	FISTA	Tikhonov	IRLS	ADMM	FISTA
AC	0.8570	0.9394	0.9577	**0.9736**	0.7200	0.9086	0.9107	**0.9299**
PC	0.9997	0.9997	**0.9998**	**0.9998**	0.997	**0.9996**	**0.9996**	**0.9996**
PSNR	76.1785	76.9921	78.9364	**79.0713**	40.5900	66.3472	75.0008	**76.2553**

### 5.4. Experiments with phantom data

**Fig. 11:**
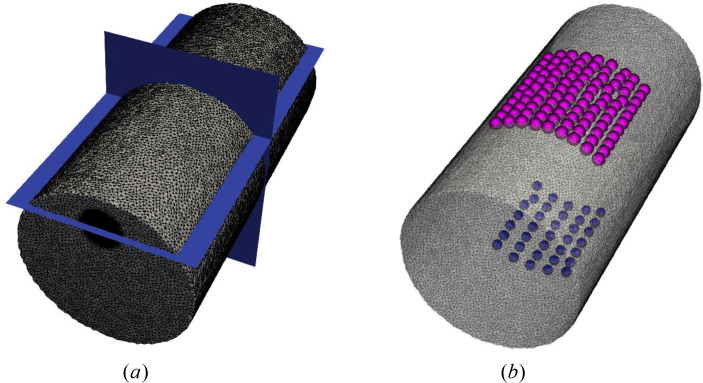
(a): Illustration of the overall distribution of slices. (b): Distribution of sources and detectors.

**Fig. 12:**
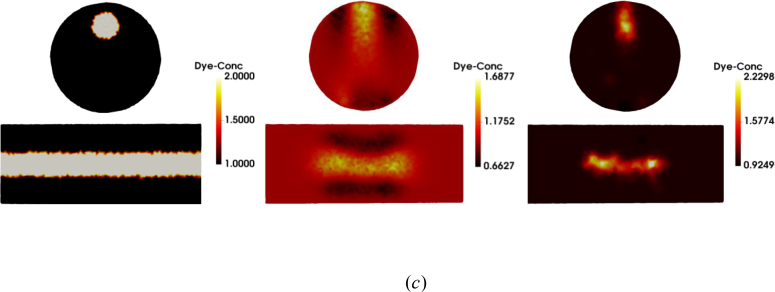
Ground truth and reconstruction results with different regularizations. From left to right: ground truth; results with L_2_ regularization; results with L_1_ regularization using FISTA algorithm.

To evaluate the proposed algorithms on real experimental data, a multispectral, non-contact CW-DOT system designed for hand imaging [64,65] was used to image a solid plastic cylindrical phantom (INO, Quebec, Canada) of radius 12.3mm and length 50mm. Boundary data was collected at five wavelengths (650nm, 710nm, 730nm 830nm and 930nm), in a transmission setup with a 7 × 5 grid of source positions on the underside of the phantom and a 11 × 9 grid of virtual detectors on top, displayed in Fig. 11(b). The spatially constant, but spectrally varying optical properties of the phantom were measured previously in time resolved experiments [66]. The absorbing dye within the phantom was treated as a chromophore that has unit concentration in the bulk of the phantom, the extinction coefficient of which was modelled by the measured absorption coefficient. A heterogeneous version of the phantom was imaged which contained a cylindrical rod of radius 3mm and length 50mm, at a depth of 5mm (Fig. 11(a)). The rod has twice the absorption coefficient of the bulk phantom which provides a 2:1 contrast in dye concentration compared to background (Fig. 12 left). A homogeneous version was also imaged, enabling calibration of the model/data mismatch and any source or detector coupling variation. The mesh, as shown in Fig. 11(a), consists of 85,205 nodes and 451,821 linear tetrahedral elements.

Ground truth data and images reconstructed with L_2_ and L_1_ methods are shown in Fig. 12 respectively. The experiments described in the previous sections showed that the particular choice of L_1_ method makes only a very small difference to the quality of the reconstruction, but there are very large differences in computational efficiency, with FISTA being far more efficient in this domain because of its superior ability to deal with large problems (as will be shown in section 5.5). Therefore in this experiment, we only use FISTA as the L_1_ solver. It can be clearly observed from Fig. 12 that L_2_ regularization over-smooths the reconstructed images which have much lower image contrast than the ground truth. Some artefacts can be seen in the source and detector areas. We note that only the central region can be reconstructed in both cases because the sources and detectors are confined to this region, with very low sensitivity away from the centre. The image contrast reconstructed by L_1_ regularization is much closer to the ground truth but with more compact results. We calculate the three evaluation metrics in the volume of illumination (Table 4) and these support the same conclusions.

**Table 4:** Evaluation of L_1_ and L_2_ regularization methods for reconstruction of a single rod inclusion in a tissue-simulating phantom.

	L_2_ Regularization	L_1_ Regularization with FISTA
AC	0.7187	1.1387
PC	0.6617	0.7263
PSNR	13.7472	13.8821

### 5.5. Comparison of CPU time consumed in the inverse model

**Fig. 13:**
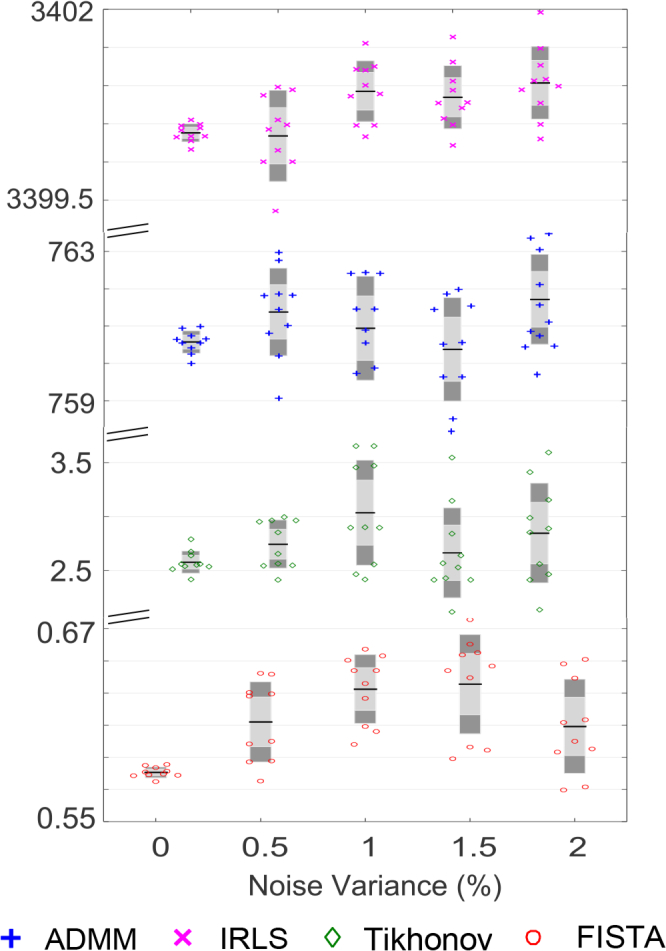
Total CPU time consumed in the experiments described in section 5.2

We now compare the computational efficiency of the proposed methods. All experiments are performed using Matlab 2013a (Mathworks, Natick, USA) on a Windows 7 (Microsoft, Redmond, USA) platform with an Intel Core CPU i7-6700 at 3.40GHz and 64.0GB memory. The simulated experiments described in section 5.2 were used to perform this comparison. CPU times used in the inverse procedure only are measured. We run each method over ten realisations of noise at each of five noise levels to obtain reliable statistics. Figure 13 shows the CPU time consumed for the four different methods (Tikhonov, IRLS, ADMM, FISTA). In order to display the recorded times from ten repetitions clearly, CPU times for one iteration of the outer loop of the reconstruction algorithm are shown in Fig. 13. Total CPU times for iteration to convergence are given in Table 5.

**Table 5:** Total CPU time(s) consumed in the inverse model for the experiments described in section 5.2

Evaluation metric	CPU time (Mean±SD)				
Chromophore	0%	0.5%	1%	1.5%	2%
Tikhonov	42.7902±0.211	33.4124±1.120	35.4658±1.399	33.4156±1.607	14.5622±1.463
IRLS	10171.96±0.019	10171.95±1.414	6823.03±0.896	6821.99±0.912	6822.01±1.274
ADMM	9100.34±0.508	7444.31±1.373	6195.01±1.583	4193.57±1.442	2892.30±1.200
FISTA	**2.532**±**0.092**	**1.964**±**0.193**	**1.844**±**0.154**	**1.850**±**0.218**	**1.927**±**0.307**

FISTA is clearly the fastest L_1_ regularization method amongst those considered here, and it is faster even than Tikhonov regularization which does not use an inner iteration. FISTA only involves the computation of J^T^J which is much more computationally efficient than the computation of JJ^T^. IRLS and ADMM are substantially slower because they require an inner iteration and inversion/multiplication of large matrices.

## 6. Conclusion

In this paper, a new model for spectrally constrained DOT reconstruction with L_1_ regularization is proposed. Numerical experiments showed that compared to the L_2_-norm, L_1_ regularization can reduce crosstalk and maintain image contrast by inducing sparsity. These findings were tested on real experimental data using a tissue-simulating phantom and similar results were found. Although all L_1_-based methods perform similarly in terms of reconstruction quality, FISTA performs marginally better than ADMM and IRLS by the measures of AC, PC, and PSNR, and is far more computationally efficient as it avoids direct matrix inversion and large matrix-matrix multiplications.

The contributions of this paper can be summarized as follows: 1) It is the first time that L_1_ regularization methods and spectrally constrained DOT methods have been used together and it is their combination (i.e. spectral-L_1_ model) that is original. We have given detailed descriptions of how this can be done, and performed systematic comparisons of the performance and efficiency of the different methods on both simulated and real data. We are not aware of any previously published work that proposes such a model and performs such a detailed analysis of its performance; 2) We have developed a method to automatically select the regularization parameters. This is based on the L-curve method but is modified for efficient application in this use-case; 3) We have conducted extensive numerical experiments on simulated data and on real experimental data, and have performed comprehensive and robust qualitative and quantitative evaluations. To the best of our knowledge, this is the first systematic study in the area of spectral DOT reconstruction to perform such a comprehensive evaluation.
